# Numerical investigation of a reaction-diffusion model used for rumor spreading in a ‘street’

**DOI:** 10.1371/journal.pone.0339059

**Published:** 2026-02-20

**Authors:** Feiyun Pei, Yamin Du

**Affiliations:** School of Economics and Management, Huainan Normal University, Huainan, China; Federal University of Pernambuco: Universidade Federal de Pernambuco, BRAZIL

## Abstract

In this paper, a reaction-diffusion model is proposed to describe the dynamics of rumor propagation among ignorant (who have not heard the rumor and are susceptible to be informed), spreader (who are spreading the rumor) and stifler (who know the rumor but that are no longer spreading it). The rumor is assumed to spread on a one-dimensional area called ‘street’. Numerical simulation is used to investigate the evolution of these three groups. The effects of the coefficients in this model, including the spreading rate α, decay rate β and self-diffusion coefficients ($D_{\text{1}}$, $D_{\text{2}}$ and $D_{\text{3}}$), are discussed. Our conclusions have the potential to explain phenomena in financial markets, information dissemination, communication networks, replicated database maintenance and disease transmission.

## 1. Introduction

In the past decades, with the development of information technology, the diffusion of the rumor is much more rapid and wide than the truth [1]. Rumor spreading [2] becomes a very common research topic in today’s society, which plays a significant role in a variety of human affairs. The content of rumors can range from gossip to propaganda and marketing material, and has a significant impact on the financial markets [3,4]. Rumor-like mechanisms form the basis for the phenomena of viral marketing, where companies exploit social networks of their customers on the Internet in order to promote their products via the so-called ‘word-of email’ and ‘word-of-web’ [5]. Several methods, such as complex networks [5–7], are used by researchers to understand the dynamics of the rumor spreading. Besides these methods, Reaction-diffusion equations [8,9], a nonlinear evolution equation varying with time and space, can be analyzed by means of analytical and numerical methods from the theory of partial differential equations and dynamical systems. It has been widely used as models for biology and medicine [10], epidemiology [11] and social sciences [12,13], which could be used to describe the process of rumor spreading.

To simplify the model, we assume the rumor spreads on a one-dimensional region representing a ‘street’. Similar model, named crimo–taxis [14], is proposed by Epstein to deal with urban crime dynamics. In this paper, Reaction-diffusion model is proposed to describe the rumor spread among the human population distributed at ‘street’ position x at time t. This nonlinear reaction-diffusion model is described in section 2. In section 3, numerical results are given to perform the evolution of rumor for different personnel distribution. On the basis of numerical calculation, we discuss the effects of spreading rate, spreading decay and diffusion coefficients on the spreading of rumor, which is given in section 4. Finally, the conclusion is given in section 5.

## 2. Nonlinear reaction-diffusion model

Rumor can be viewed as an ‘infection of the mind’, whose spreading could be expressed as social interactions in human population. The human population is divided into 3 groups [15], i.e., ignorant, spreader and stifler. Ignorant is the person who has not heard the rumor and susceptible to be informed. Spreader is the person who spreads rumor. Stifler knows the rumor, but no longer spreads it, who corresponds to death, isolation or immunity in the real world.

The spreading process is assumed through direct contact between the spreaders and others in the population. When a spreader encounters an ignorant, the ignorant has a certain spreading rate *α* of becoming a new spreader, while the identity of the spreader unchanged. This process corresponds to the increasing of spreader. When a spreader encounters a spreader or a stifler, they will exchange information which may lead them to become aware of rumor and lose the motivation to spread rumor. It leads to a certain rate *β* that the spreader may transform into a stifler. This process corresponds to the decay of the spreading process.

In a closed system, we define u(x,t), v(x,t) and w(x,t) as the normalized number of ignorant, spreader and stifler at normalized ‘street’ position x (0≤x≤1) at time t, respectively. The normalization condition is shown as equation 1, which indicates that the total number of people remains constant (unit 1) at any time in this closed system.


$$\int_{\text{0}}^{\text{1}}\left[u(x,t)+v(x,t)+w(x,t)\right]\cdot dx=\text{1}$$ (1)

Based on Ref. [7] and considering diffusion in one-dimensional space, we incorporate diffusion terms into the original model; the evolution of the three densities is then governed by the following set of coupled differential equations:


$$\frac{\partial u(x,t)}{dt}=-\alpha \cdot u(x,t)\cdot v(x,t)+D_{\text{1}}\cdot \frac{\partial^{\text{2}}u(x,t)}{{\partial x}^{\text{2}}}$$ (2)

$$\frac{\partial v(x,t)}{dt}=\alpha \cdot u(x,t)\cdot v(x,t)-\beta \cdot v(x,t)\cdot \left[v(x,t)+w(x,t)\right]+D_{\text{2}}\cdot \frac{\partial^{\text{2}}v(x,t)}{{\partial x}^{\text{2}}}$$ (3)

$$\frac{\partial w(x,t)}{dt}=\beta \cdot v(x,t)\cdot \left[v(x,t)+w(x,t)\right]+D_{\text{3}}\cdot \frac{\partial^{\text{2}}w(x,t)}{{\partial x}^{\text{2}}}$$ (4)

The above equations state that the density of spreaders increases at a rate proportional to the spreading rate α. On the other hand, the annihilation mechanism considers that spreaders decay into the stifler class at a rate β times the sum of the density of spreaders and stiflers, i.e., the density of non-ignorants at time t. $D_{\text{1}}$, $D_{\text{2}}$ and $D_{\text{3}}$ are self-diffusion coefficients for ignorant, spreader and stifler, respectively. Here, we do not consider the influence of other density distributions on density diffusion, and the cross-diffusion coefficients are set to 0. Equation 2–4 tell us that the densities of ignorant, spreader and stifler at (x,t) are not only determined by the interaction among themselves, but also influenced by their respective diffusion effects.

## 3. Numerical simulation

In terms of space, the ‘street’ (0≤x≤1) has been divided evenly into 100 segments (yielding 101 nodal points). The ignorants, spreaders and stiflers are placed at these grid points according to a certain distribution. The processes of spreading and decay occur at the grid points, and the process of diffusion occurs between grid points. In terms of time, each iteration is equivalent to one time of spreading and/or decay, corresponding to the pass of time. The central difference scheme for u(x,t) at point $\text{x}={\text{x}}_{\text{i}}$ for the n-th iteration is given as equation 5.


$${\;\frac{\partial^{\text{2}}u(x,t)}{{\partial x}^{\text{2}}}|}_{x=x_{i}}=\frac{u_{i+\text{1}}^{n}-\text{2}u_{i}^{n}+u_{i-\text{1}}^{n}}{(\Delta x{)}^{\text{2}}}$$ (5)

where ${\text{u}}_{\text{i}}^{\text{n}}=\text{u}\left({\text{x}}_{\text{i}},\text{n}\right)$, and Δx is the uniform spatial step size. The same treatment is applied to v(x,t) and w(x,t).

In our simulation, assuming the initial conditions $\int_{\text{0}}^{\text{1}}u(x,\text{0})\cdot dx=\text{0}.\text{8}$, $\int_{\text{0}}^{\text{1}}v(x,\text{0})\cdot dx=\text{0}.\text{2}$ and w(x,0)=0. the values of spreading rate α, decay rate β, and self-diffusion coefficients ($D_{\text{1}},\;D_{\text{2}}\;and\;D_{\text{3}}$) are assumed to be constants due to simplification. In this section, we assume α=0.8, β=0.4, $D_{\text{1}}=D_{\text{2}}=D_{\text{3}}=\text{1}.\text{0}e-\text{6}$, and investigates the evolution of rumor for different initial population distribution forms.

### 3.1 Uniform distribution

Firstly, using the simplest distribution, it is assumed that ignorants (80% of the sample) and spreaders (20% of the sample) are evenly distributed in the 101 divided grid points, shown as equation 6.


$$\left\{ \begin{array}{c} @ru\left(x_{i},\text{0}\right)=\frac{\text{8}\text{0}\%}{\text{1}\text{0}\text{1}}\mathrm{\backslash vspace}1mm\\ v\left(x_{i},\text{0}\right)=\frac{\text{2}\text{0}\%}{\text{1}\text{0}\text{1}} \end{array}\;\right.\;,\;\left(\text{0}\leq x_{i}\leq \text{1}\;\&\;\text{1}\leq i\leq \text{101}\right)$$ (6)

Due to the uniform distribution, $\frac{\partial^{\text{2}}u(x,\text{0})}{{\partial x}^{\text{2}}}=\frac{\partial^{\text{2}}v(x,\text{0})}{{\partial x}^{\text{2}}}=\frac{\partial^{\text{2}}w(x,\text{0})}{{\partial x}^{\text{2}}}=\text{0}$. The values of $\alpha ,\;\beta ,\;D_{\text{1}},\;D_{\text{2}}\;and\;D_{\text{3}}$ are assumed to be constants for different grids, the set of equation 2–4 is converted into equation 7, which is similar to the equation 2–4 in reference [7].


$$\left\{ \begin{array}{c} @r\frac{\partial u(x,t)}{dt}=-\alpha \cdot u(x,t)\cdot v(x,t)\;\\ \frac{\partial v(x,t)}{dt}=\alpha \cdot u(x,t)\cdot v(x,t)-\beta \cdot v(x,t)\cdot \left[v(x,t)+w(x,t)\right]\\ \frac{\partial w(x,t)}{dt}=\beta \cdot v(x,t)\cdot \left[v(x,t)+w(x,t)\right]\; \end{array}\right.\;$$ (7)

**Fig 1:**
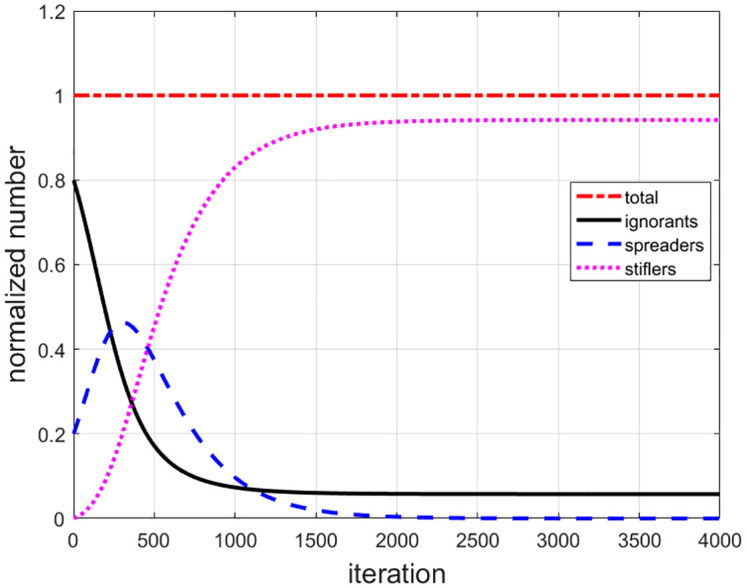
The temporal evolution of total number of ignorants (black line), spreaders (blue dashed line) and stiflers (magenta dotted line) for uniform distribution. During the evolution, the total number of ignorants, spreaders and stiflers is unit 1 (red dash-dot line).

Fig 1 shows the temporal evolution of number of ignorants (black line), spreaders (blue dashed line) and stiflers (magenta dotted line). The spreaders increase to maximum value of 46.29% at t=302, and then reduce to 1% at t=1705, and finally approach 0 slowly and smoothly. Because of rumor spreading, the ignorants decrease to (5.76±1 at about t=1127. Due to the decay mechanism, spreaders convert to the stiflers leading to an increase in stiflers and a decrease in spreaders. Stiflers increase to the stable value of about (94.24±1 at about t=1746. We found that, until the end, there will still be about 5.76% of ignorants who have not been infected, about 94.24% of stiflers who are immunized to rumors, and near 0 (0.0005%) spreaders who remain active after a sufficiently long time (t=4000). During the evolution, the total number of ignorants, spreaders and stiflers is unit 1 (red dash-dot line).

**Fig 2:**
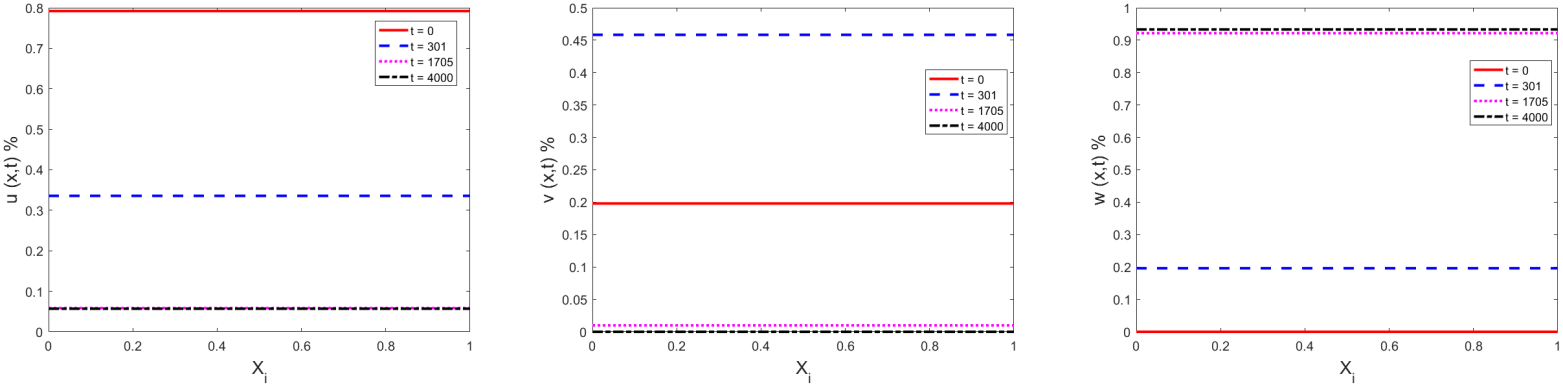
Space distribution evolutions of ignorants (left: 𝐮(x,t)), spreaders (middle: 𝐯(x,t)) and stiflers (right: 𝐰(x,t)) located at 101 grid points at 𝐭=0, 𝐭=301, 𝐭=1705 and 𝐭=4000.

Fig 2 gives the space distribution of ignorants (left), spreaders (middle) and stiflers (right) at t=0, t=301, t=1705 and t=4000, respectively. As mentioned before, the diffusion terms of equations (2)–(4) has disappeared for uniform distribution at t=0, and the distributions of ignorants, spreaders and stiflers are flat during the evolution. The distribution of t=1705 and t=4000 basically overlap, and the distribution has basically stabilized during this period.

### 3.2 Gaussian distribution

In the real world, people always gather together, corresponding to a large number of personnel at the center and a small number of personnel at the edge. In this paper, Gaussian distribution is used to describe this characteristic, shown as equation 8.


$$\left\{ \begin{array}{c} @lu\left(x_{i},\text{0}\right)=\frac{\text{8}\text{0}\%}{\sqrt{\text{2}\pi }\sigma_{\text{1}}}\text{exp}\left[-\frac{{\left(x_{i}-\mu_{\text{1}}\right)}^{\text{2}}}{\text{2}\sigma_{\text{1}}^{\text{2}}}\right]/sum\left(\vec{u}(x,\text{0})\right)\mathrm{\backslash vspace}1mm\\ v\left(x_{i},\text{0}\right)=\frac{\text{2}\text{0}\%}{\sqrt{\text{2}\pi }\sigma_{\text{2}}}\text{exp}\left[-\frac{{\left(x_{i}-\mu_{\text{2}}\right)}^{\text{2}}}{\text{2}\sigma_{\text{2}}^{\text{2}}}\right]/sum\left(\vec{v}(x,\text{0})\right) \end{array}\;\right.\;,\;\left(\text{0}\leq x_{i}\leq \text{1}\;\&\;\text{1}\leq i\leq \text{101}\right)$$ (8)

where $sum\left(\vec{u}(x,\text{0})\right)=\sum_{i=\text{1}}^{\text{1}\text{0}\text{1}}\frac{\text{1}}{\sqrt{\text{2}\pi }\sigma_{\text{1}}}\text{exp}\left[-\frac{{\left(x_{i}-\mu_{\text{1}}\right)}^{\text{2}}}{\text{2}\sigma_{\text{1}}^{\text{2}}}\right]$ and $sum\left(\vec{v}(x,\text{0})\right)=\sum_{i=\text{1}}^{\text{1}\text{0}\text{1}}\frac{\text{1}}{\sqrt{\text{2}\pi }\sigma_{\text{2}}}\text{exp}\left[-\frac{{\left(x_{i}-\mu_{\text{2}}\right)}^{\text{2}}}{\text{2}\sigma_{\text{2}}^{\text{2}}}\right]$. We consider the ignorants gather in the center of the ‘street’ by defining $\mu_{\text{1}}=\text{0}.\text{5}$. Because the area inside x∈(μ−2σ,  μ+2σ) is 95.45% for Gaussian distribution, we define $\sigma_{\text{1}}=\text{0}.\text{25}$ to describe the initial group of the ignorants. As σ decreases, the Gaussian distribution becomes more concentrated. Assuming the initial spreaders concentrated in a narrow area by defining $\sigma_{\text{2}}=\text{0}.\text{005}$.

**Fig 3:**
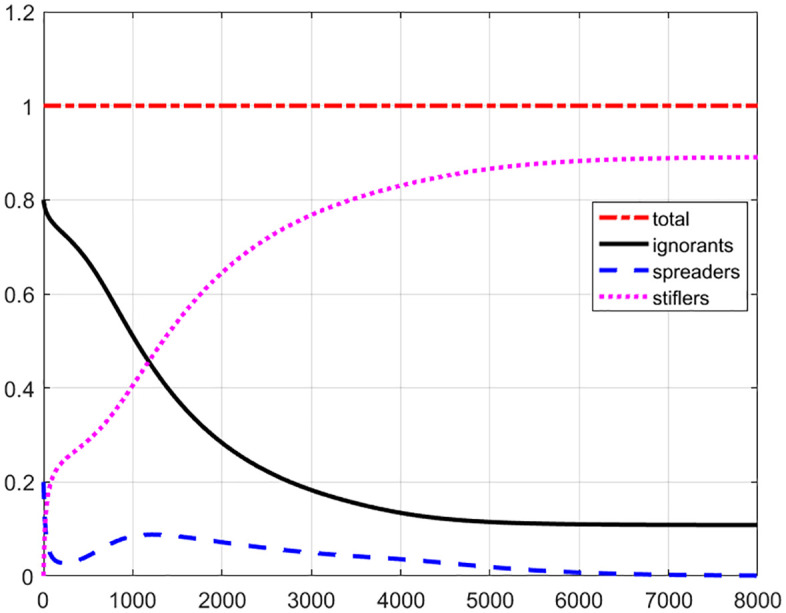
The temporal evolution of total number of ignorants (black line), spreaders (blue dashed line) and stiflers (magenta dotted line) for Gaussian distribution with initial center rumor. During the evolution, the total number of ignorants, spreaders and stiflers is unit 1 (red dash-dot line).

For the first case, we consider the initial rumors mainly generate in the center of ‘street’ by defining $\mu_{\text{2}}=\text{0}.\text{5}$. The temporal evolution of total number of ignorants, spreaders and stiflers are given as Fig 3. The trajectory of spreaders number is interesting. Unlike case of uniform distribution (section 3.1), the number of spreaders decreases quick to 2.77% at t=230, then increase relatively slowly to a regional maximum value 8.80% at t=1229, and finally decrease to close to 0 (0.09%) at t=8000. The ignorants number decrease to a stable value 10.86% at t=8000, while the stiflers number increase to a stable value 89.04% at t=8000. Comparing to uniform distribution, it takes more time to reach a stable value, and almost 5.1% more ignorants do not convert to spreaders, and maintain their identity as ignorant individuals throughout the entire evolutionary process.

**Fig 4:**
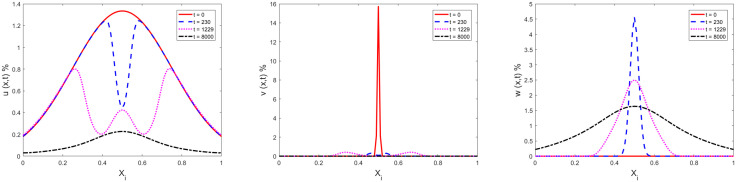
Space distribution evolutions of ignorants (left: 𝐮(x,t)), spreaders (middle: 𝐯(x,t)) and stiflers (right: 𝐰(x,t)) located at 101 grid points at 𝐭=0, 𝐭=230, 𝐭=1229 and 𝐭=8000.

The space distributions of ignorants (left), spreaders (middle) and stiflers (right) at t=0, t=230, t=1229 and t=8000 are shown in Fig 4, respectively. The initial Gaussian distributions of ignorants and spreaders are shown as red line (t=0). The maximum density of ignorants and spreaders located at the position x=0.5, and intense interactions of spreading and decay occur at the x=0.5. it causes a hole of ignorants density and a peak of stiflers at t=230. Then, the diffusion of ignorants, spreaders and stiflers are beginning to dominate. The hole of ignorants density begin to recovery, and the peak of stiflers begin to flat.

**Fig 5:**
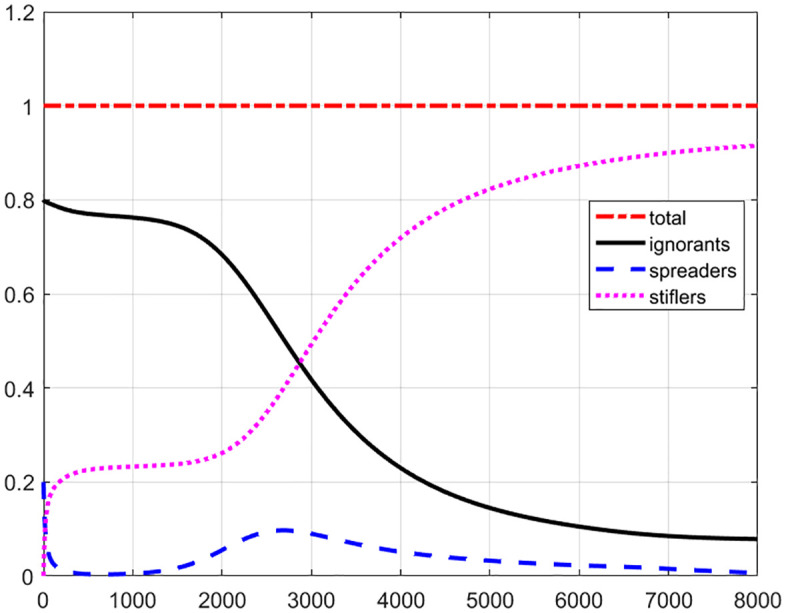
The temporal evolution of total number of ignorants (black line), spreaders (blue dashed line) and stiflers (magenta dotted line) for Gaussian distribution with initial edge rumor. During the evolution, the total number of ignorants, spreaders and stiflers is unit 1 (red dash-dot line).

For the second case, we consider the initial rumors is mainly located at the edge of ‘street’ by defining $\mu_{\text{2}}=\text{0}.\text{05}$. The temporal evolution of total number of ignorants, spreaders and stiflers are given as Fig 5. The number of spreaders drops rapidly before t=200, and decrease slowly to the regional minimum value 0.34% at t=649. It seems the rumors have been eliminated. But then, the trends changes, the spreaders increase to regional maximum value 9.71% at t=2674, as if rumors were reigniting. Finally, the number deceases slowly to 0.65% at t=8000. At the same time, the ignorants number decrease to a stable value 7.85%, while the stiflers number increase to a stable value 91.49%, which is close to the first case.

**Fig 6:**
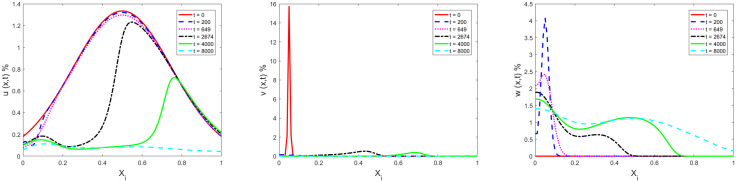
Space distribution evolutions of ignorants (left: 𝐮(x,t)), spreaders (middle: 𝐯(x,t)) and stiflers (right: 𝐰(x,t)) located at 101 grid points at 𝐭=0, 𝐭=200, 𝐭=649, 𝐭=2674, 𝐭=4000 and 𝐭=8000.

The space distributions of ignorants (left), spreaders (middle) and stiflers (right) at t=0, t=200, t=649, t=2674, t=4000 and t=8000 are shown in Fig 6, respectively. Due to the initial distribution of spreader, the effects of spreading and decay are occurred at the edge of ‘street’ locally. At the edge of the ‘street’, many ignorants change to the spreaders. And then, the original and newly converted spreaders change to the stiflers, which is responsible to the peak of stiflers at t=200. If there are no diffusion terms $\left(D_{\text{1}}=D_{\text{2}}=D_{\text{3}}=\text{0}\right)$, the spreaders will be converted to stiflers locally and eradicated finally. Due to the diffusion terms, the remaining spreaders diffuse to the new place and develop the new spreaders, which explains the increasing of spreaders between t=649 and t=2674. At t=2674, shown as left figure in Fig 6, most ignorants are located on the right side of ‘street’. The density of remaining ignorants, who may be converted to spreaders, start to decrease. It explains the reduce of spreaders since t=2674. After t=2674, with the diffusion of spreaders, the right side of ignorants change to the spreaders, and then convert to stiflers. It corresponds to the increasing of stiflers and decreasing of ignorants in the final stage.

## 4. The effect of the coefficients

For the set of equations 2–4, the coefficients include the spreading rate α and decay rate β, which represent the interaction between the ignorants, spreaders and stiflers, while self-diffusion coefficients $D_{\text{1}}$, $D_{\text{2}}$ and $D_{\text{3}}$, which represent the diffusion of ignorants, spreaders and stiflers along the ‘street’. In the introduction of the section 3, we found that uniform distribution naturally eliminates the diffusion terms, which are related to self-diffusion coefficients $D_{\text{1}}$, $D_{\text{2}}$ and $D_{\text{3}}$. So, we analysis the effect of the spreading rate α and decay rate β with uniform distribution at first.

**Fig 7:**
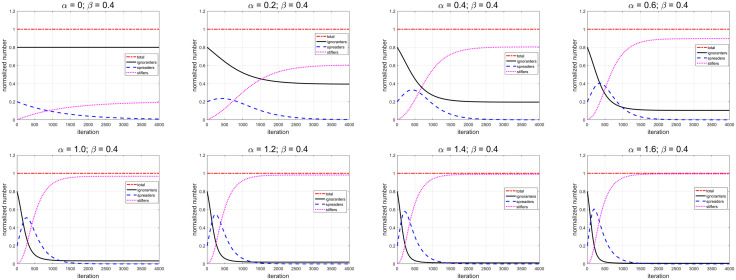
Using uniform distribution, the temporal evolution of total number of ignorants (black line), spreaders (blue dashed line) and stiflers (magenta dotted line) for different spreading rate α.

Firstly, we scan the spreading rate α from 0 to 1.6, while the decay rate β is constant (0.4), shown as Fig 7. The key results are listed in Table 1. The maximum value of spreaders increases with α increase. The time, when the spreaders reach its maximum, would increase with the growing of α for α<0.4, and then decrease with the growing of α. For a smaller α, it takes longer time to reach the stable value. It means the course of rumor spreading will last long time. At the same time, fewer ignorants convert to spreaders, and change to the stiflers finally. The number of ignorants is relative higher, while the number of stiflers is relative lower. It means the course of rumor spreading is of low intensity.

**Table 1:** Key results of the evolutions for ignorants, spreaders and stiflers with different spreading rate α (uniform distribution).

α	β	Maximum value		$t_{end}=4000$		
		𝐭	${𝐯}_{max}$	$𝐬𝐮𝐦\left(u\left(x,t_{end}\right)\right)$	$𝐬𝐮𝐦\left(v\left(x,t_{end}\right)\right)$	$𝐬𝐮𝐦\left(w\left(x,t_{end}\right)\right)$
**0.00**	0.40	1	20%	80%	0.84%	19.16%
**0.20**	0.40	395	23.54%	39.42%	0.22%	60.36%
**0.40**	0.40	417	33.03%	19.54%	0.01%	80.45%
**0.60**	0.40	351	40.50%	10.38%	0.002%	89.62%
**0.80**	0.40	302	46.29%	5.76%	0.0005%	94.24%
**1.00**	0.40	265	50.90%	3.29%	0.0002%	96.71%
**1.20**	0.40	235	54.66%	1.92%	0.0001%	98.08%
**1.40**	0.40	215	57.81%	1.13%	0.00006%	98.87%
**1.60**	0.40	196	60.48%	0.67%	0.00004%	99.33%

**Fig 8:**
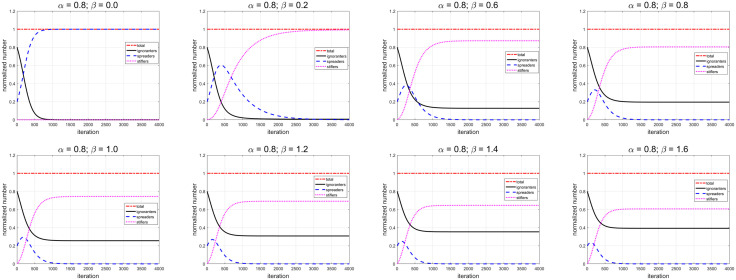
Using uniform distribution, the temporal evolution of total number of ignorants (black line), spreaders (blue dashed line) and stiflers (magenta dotted line) for different decay rate β.

Then, we scan the decay rate β from 0 to 1.6, while the spreading rate α is constant (0.8), shown as Fig 8. The key results are listed in Table 2. When β=0, it means there is no decay effect which convert the spreaders into stiflers. As a result, all ignorants change to the spreaders. There is no ignorants and stiflers in the ‘street’ at last. As β increases, the time which the spreaders required to reach the maximum value reduce, and the maximum value of spreaders decreases. It means the peak of rumor spreading comes early and the degree is low for large β. At $t_{end}=\text{4}\text{0}\text{0}\text{0}$, due to the suppressive effect of β, the higher value of β leads to the more ignorants and the less stiflers. It means higher β protects more ignorants from rumors. Thereby, it reduces the stiflers who have been ‘infected’ by rumors but no longer spread it. These stiflers may be the victims of rumors.

**Table 2:** Key results of the evolutions for ignorants, spreaders and stiflers with different decay rate β (uniform distribution).

α	β	Maximum value		$t_{end}=4000$		
		𝐭	${𝐯}_{max}$	$𝐬𝐮𝐦\left(u\left(x,t_{end}\right)\right)$	$𝐬𝐮𝐦\left(v\left(x,t_{end}\right)\right)$	$𝐬𝐮𝐦\left(w\left(x,t_{end}\right)\right)$
0.80	0.00	N/A	N/A	0%	100%	0%
0.80	0.20	388	60.41%	0.68%	0.094%	99.23%
0.80	0.40	302	46.29%	5.76%	0.0005%	94.24%
0.80	0.60	249	38.25%	12.73%	0.00001%	87.27%
0.80	0.80	207	33.05%	19.51%	9.7e-9	80.49%
0.80	1.00	176	29.47%	25.57%	1.1e-9	74.43%
0.80	1.20	147	26.89%	30.83%	1.7e-10	69.17%
0.80	1.40	122	24.99%	35.37%	3.2e-11	64.63%
0.80	1.60	102	23.57%	39.28%	6.8e-12	60.72%

The effects of self-diffusion coefficients $D_{\text{1}}$, $D_{\text{2}}$ and $D_{\text{3}}$ are given by the case that initial rumors spreaders are mainly located at the edge. For comparison purpose, the spreading rate α=0.80 and the decay rate β=0.40. We change the self-diffusion coefficients $D_{\text{1}}$, $D_{\text{2}}$ and $D_{\text{3}}$ one by one, and observe the difference between their evolutions, shown as Table 3. Case 1, as a reference, corresponds to Fig 5 and 6 in section 3.

**Table 3:** Key results of the evolutions for ignorants, spreaders and stiflers with different self-diffusion coefficients $D_{\text{1}}$, $D_{\text{2}}$ and $D_{\text{3}}$ (Gaussian distributions).

No.	${𝐃}_{1}$	${𝐃}_{2}$	${𝐃}_{3}$	Regional min		Regional max		${𝐭}_{end}=8000$		
				𝐭	${𝐯}_{min}$	𝐭	${𝐯}_{max}$	𝐬𝐮𝐦(u)	𝐬𝐮𝐦(v)	𝐬𝐮𝐦(w)
**1**	1.0e-6	1.0e-6	1.0e-6	649	0.34%	2674	9.71%	7.85%	0.66%	91.49%
**2**	**5.0e-6**	1.0e-6	1.0e-6	651	0.34%	2711	8.65%	6.77%	0.67%	92.56%
**3**	1.0e-6	**5.0e-6**	1.0e-6	426	2.27%	1534	17.39%	6.05%	0.03%	93.92%
**4**	1.0e-6	1.0e-6	**5.0e-6**	962	0.15%	2817	9.82%	12.40%	0.25%	87.35%

**Fig 9:**
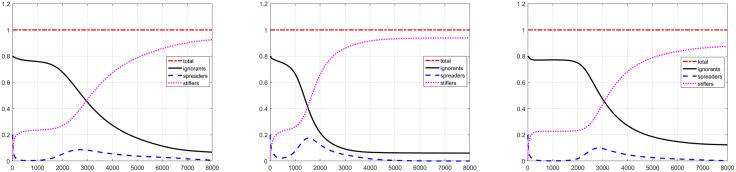
The temporal evolution of number for three group corresponding to case 2 (left), case 3 (middle) and case 4 (right) in Table 3.

Only increasing the self-diffusion coefficient of ignorants $D_{\text{1}}$ (case 2 in Table 3), we could find that the key results, including the regional minimum & maximum and numbet of each group at last time, are similar to the case 1. The evolutions of total number for three groups are given in Fig 9 (left), which is almost same to Fig 5 for case 1. The effect of $D_{\text{1}}$ on the evolutions of spreaders and stiflers is not very significant.

Case 3 only considers the effect of self-diffusion coefficient $D_{\text{2}}$. Shown as Fig 9 (middle), it directly affects the evolution of spreaders, which causes the spreaders number rapidly ramp down to a regional minimum value at first stage, then ramp up quickly to a regional maximum value, and slowly reduces to approach a fixed value. The regional minimum value and maximum value are higher than the data of case 1, which means an intense spreading for case 3. Compared to case 1, it approaches the fixed value faster, which means the time required to spread the rumor is much shorter. Correspondingly, the process of ignorants becoming stiflers is also faster, while the decrease in remain ignorant individuals is not very significant.

In case 4, we only enhance the mobility of stiflers by increasing $D_{\text{3}}$. Shown as Fig 9 (right), the spreaders reduce quickly at the early stage, Later, it enters a process of slow reduction and then recovery, which is very smooth and lasts for a very long time. This process corresponds to the low intensity spread of rumors, rather than the end of the rumor. Since t=2000, the growth of spreader has reached a small peak, and then trends towards a fixed value. We found that the remaining ignorants are much larger than other cases.

## 5. Conclusion

In this paper, a reaction-diffusion model, which consist of three coupled reaction-diffusion equations involving self-diffusion of ignorants, spreaders and stiflers, is proposed to simulate the rumor spreading on a one-dimensional ‘street’. By this model, we have analyzed the dynamics of rumors spreading for population in Uniform distribution form and Gaussian distribution form. Besides temporal evolution of ignorants, spreaders and stiflers, we also give their space distributions along the ‘street’ at some important time-slices. The results of numerical simulation provide an intuitive process of rumor propagation.

Based on the Numerical investigation of reaction-diffusion model, we explore the effect of spreading rate α, decay rate β and self-diffusion coefficients $\left(D_{\text{1}},\;D_{\text{2}},\;D_{\text{3}}\right)$ on the dynamics of rumors spreading. (1) Larger spreading rate α causes a shorter rumor spreading process and faster propagation process. The number of ignorants who are not ‘infected’ by rumors is smaller finally. (2) larger decay rate β results in a shorter rumor spreading process and more moderate propagation process. The number of ignorants who are not ‘infected’ by rumors is larger at last. (3) Self-diffusion coefficient of ignorants $D_{\text{1}}$ seems to have little impact on the evolutions of spreaders and stiflers. It mainly changes the spatial distribution of ignorants. (4) Self-diffusion coefficient of spreaders $D_{\text{2}}$, corresponding to diffusion of spreaders, makes the propagation process fast. Larger $D_{\text{2}}$ leads to more spreaders at each time-slice in the early stage of rumors spread. The remaining ignorants reduce slightly from 7.85% to 6.05% when $D_{\text{2}}$ changes from 1.0 × 10^−6^ to 5.0 × 10^−6^. (5) Self-diffusion coefficient of stiflers $D_{\text{3}}$ causes long-term low-density rumor spread. As $D_{\text{3}}$ increases, more ignorants can avoid the influence of rumors and thus maintain their identity as ignorants.

The more interesting work is to make more careful improvements to the model to adapt to the spread of rumors in reality. For example, spreading rate α and decay rate β could be modelled as a function of (x, t) to indicate that the interaction rate varies in different environments. Some enlightening results have been found in reference 5–7. In some real situations, the diffusions of ignorants, spreaders and stiflers are not only influenced by their own distribution, but also by other distributions. The cross-diffusion terms can be considered in our future research. Our research aims to have the ability to explain more phenomena in financial markets, information dissemination, communication networks, replication database maintenance, and disease transmission.
